# Corticotropin releasing hormone receptor *CRHR1* gene is associated with tianeptine antidepressant response in a large sample of outpatients from real-life settings

**DOI:** 10.1038/s41398-020-01067-y

**Published:** 2020-11-05

**Authors:** Nicolas Ramoz, Nicolas Hoertel, Bénédicte Nobile, Géraldine Voegeli, Ariane Nasr, Yann Le Strat, Philippe Courtet, Philip Gorwood

**Affiliations:** 1 Université de Paris, Institute of Psychiatry and Neuroscience of Paris (IPNP), INSERM U1266, Team Vulnerability of Psychiatric and Addictive Disorders, 75014 Paris, France; 2 Assistance Publique-Hôpitaux de Paris (APHP), Corentin Celton Hospital, Department of Psychiatry, 92130 Issy-les-Moulineaux, France; 3 University of Paris, Paris, France; 4 Department of Emergency Psychiatry and Acute Care, CHU Montpellier, INSERM U1061, Montpellier University, Montpellier, France; 5 GHU Paris Psychiatrie et Neurosciences, Clinique des Maladies Mentales et de l’Encéphale (CMME), Centre Hospitalier Sainte-Anne, Paris, France; 6 Service de Psychiatrie, Hôpital Louis Mourier, Assistance Publique-Hôpitaux de Paris, Colombes, France

**Keywords:** Clinical genetics, Prognostic markers

## Abstract

Polymorphisms of genes involved in the hypothalamic–pituitary–adrenocortical (HPA) axis have been associated with response to several antidepressant treatments in patients suffering of depression. These pharmacogenetics findings have been reported from independent cohorts of patients mostly treated with selective serotonin reuptake inhibitors, tricyclic antidepressant, and mirtazapine. Tianeptine, an atypical antidepressant, recently identified as a mu opioid receptor agonist, which prevents and reverses the stress induced by glucocorticoids, has been investigated in this present pharmacogenetics study. More than 3200 Caucasian outpatients with a major depressive episode (MDE) from real-life settings were herein analyzed for clinical response to tianeptine, a treatment initiated from 79.5% of the subjects, during 6–8 weeks follow-up, assessing polymorphisms targeting four genes involved in the HPA axis (*NR3C1*, *FKPB5*, *CRHR1,* and *AVPR1B*). We found a significant association (*p* < 0.001) between *CRHR1* gene variants rs878886 and rs16940665, or haplotype rs878886*C–rs16940665*T, and tianeptine antidepressant response and remission according to the hospital anxiety and depression scale. Analyses, including a structural equation model with simple mediation, suggest a moderate effect of sociodemographic characteristics and depressive disorder features on treatment response in individuals carrying the antidepressant responder allele rs8788861 (allele C). These findings suggest direct pharmacological consequences of *CRHR1* polymorphisms in the antidepressant tianeptine response and remission, in MDE patients. This study replicates the association of the *CRHR1* gene, involved in the HPA axis, with (1) a specificity attributed to treatment response, (2) a lower risk of chance finding, and in (3) an ecological situation.

## Introduction

Major depressive disorder (MDD) is usually considered as being associated with a deregulation of the hypothalamic–pituitary–adrenocortical (HPA) axis. The hyperactivity of the HPA system might be testified by an elevated neurotransmission of the corticotropin-releasing hormone (CRH/CRF) and arginine vasopressin (AVP) peptides, which stimulate the production of adrenocorticotropic hormone and cortisol, leading to a low-grade inflammation in patients with MDD^1^. Furthermore, the negative feedback is controlled by the glucocorticoid receptors (GR) and interacting proteins, like FKBP5 protein^2–5^. Interestingly, ketamine (an NMDA antagonist) has been shown to significantly improve depression symptoms within patients with treatment-resistant depression, may be partly due to its action on inflammation^6,7^. Those data support the hypothesis of HPA axis dysfunction within MDD patients and consequently the hypothesis of improving depression by acting on this system. Convergent evidence showed that antidepressant treatments may normalize or modulate the abnormal functionality of the HPA system in MDD patients^8–11^. It seems legitimate to suppose that response to antidepressant treatment could be different according to antidepressant type and genetic polymorphisms of the HPA axis.

Accordingly, several polymorphisms of genes, involved in HPA axis, have been associated with response to several antidepressant treatments. These results of pharmacogenetics were observed for several polymorphisms of *FKBP5* gene (which encodes a chaperon protein to GR) associated with clinical response in European Caucasian MDD patients to various antidepressant treatments, including selective serotonin reuptake inhibitors (SSRI), tricyclic antidepressant (TCA), and mirtazapine (drug targeting serotonergic and noradrenergic receptors)^12^. The amino acid change N363S in the glucocorticoid receptor, encoded by the single polymorphism nucleotide (SNP) rs56149945/rs6195 in the *NR3C1* gene, was investigated among 367 depressed patients having received either SSRI, TCA, or mirtazapine^13^. This *NR3C1* variant was associated with early response after 2 weeks of treatment^13^. Another pharmacogenetics association was observed between three SNPs encompassing *NR3C1* gene and response to escitalopram (SSRI) or nortriptyline (a norepinephrine reuptake inhibitor) among 760 patients of the Genome-based Therapeutic Drugs for Depression study (GENDEP study)^14^. Haplotypes of three variants of the CRH receptor type 1 *CRHR1* gene were associated with an SSRI (fluoxetine) or a TCA (desipramine), in highly anxious Mexican-American patients^15^. The pharmacogenetics association of *CRHR1* gene with response and remission was confirmed in the Sequenced Treatment Alternatives to Relieve Depression, STAR*D cohort, a sample of 1768 MDD patients treated initially by an SSRI (citalopram)^16^. In this study, polymorphisms in CRH-binding protein gene (*CRHBP*), CRH receptor 2 gene (*CRHR2*), *CRH* gene, and arginine vasopressin receptor 1A gene (*AVPR1A*) were also associated with treatment response or remission^16^.

We therefore have converging evidence that genes can be used to efficiently participate in the prediction of antidepressants response, and many of these genes are involved in the HPA regulation. Nevertheless, replication study was not systematic, although independent replication is considered as one of the most solid way to eliminate or, at least, reduce chance finding, especially for samples with modest size. Assessing various biomarkers predicting antidepressant response in a large meta-analysis, *FKBP5* gene was indeed identified as the second gene having the largest effect size reaching a significant threshold^17^, testifying in favor of a potential use in clinical practice. Nevertheless, antidepressants have variable mechanisms of action, and other antidepressants (i.e. apart from SSRI, SNRI and tricyclics) are worth being analyzed for their pharmacogenetics factors. We also need to further distinguish the (direct) pharmacogenetics effects from (intermediate) clinical variables, as many are known to be involved in treatment response (such as number of past episodes, level of anxiety, and severity of depression). Indeed, a gene involved in the HPA axis could be associated with higher vulnerability to severe, comorbid, or chronic depressive disorder. All these traits are strongly related to poorer chances of treatment response^18^. Finally, if pharmacogenetic associations are replicated and not attributed to a clinical intermediate factor, it is important to assess if such information is predictive in “real life”, meaning in a large cohort of usual outpatients (i.e. with few exclusion criteria) evaluated in an ecological situation.

Tianeptine is an antidepressant with structural similarities with TCAs, but with different pharmacological properties. Tianeptine is an atypical antidepressant because its mechanisms of action clearly challenge the monoamine hypothesis, triggering a cascade of cellular adaptations that leads to antidepressant response. Interestingly, tianeptine prevents and reverses the stress induced by glucocorticoids (through dendritic remodeling in CA3 pyramidal neurons in the hippocampus), and it increases dendritic length and branching of neurons in the amygdala^19,20^. Tianeptine is able to inhibit the dendritic remodeling caused by stress or glucocorticoids^20^. Finally, tianeptine has been shown to be a mu opioid receptor (MOR) agonist^21^ and its antidepressant effects may be mediated by this receptor^22^. This is the most interesting role of tianeptine since opioid system is known to partly regulate HPA axis. Indeed, an MOR antagonist would increase HPA axis activation^23^ and an MOR agonist would mediate this activation^24^. Clinical efficacy and tolerability of tianeptine have been demonstrated in depressed patients^25^. Our goal was thus to investigate the pharmacogenetics of tianeptine response by screening genes coding the proteins that play a major role in the HPA axis, in an ecological sample to test the predictive capacity of this set of HPA genes.

We therefore collected a sample of outpatients treated with tianeptine for a major depressive episode (MDE). More than 3200 Caucasian outpatients were analyzed for clinical response to tianeptine and assessed by the genotyping of different SNPs targeting several genes involved in the HPA axis.

## Materials and methods

### Outpatients and clinical assessment

We used a French network of psychiatrists and general physicians who already collected large samples of depressed outpatients^26,27^ to constitute a novel cohort of antidepressant treated patients, named GENESE. This time, we included in the research tools a device allowing to collect saliva in order to perform pharmacogenetic analyses. A total of 3771 outpatients were enrolled. For this pharmacogenetic study, we restricted the analyses to the 3212 Caucasians (85.18%) with available DNA (*N* = 3042, 94.71%) to reduce the risk of stratification biases at the genetic level, due to the variability of the allelic percentages of genetic variants in different ethnic groups. The sample size of 3212 Caucasian outpatients was sufficient to detect a pharmacogenetics difference of 5% between the responder and non-responder groups.

GENESE is a large prospective, naturalistic cohort of French outpatients diagnosed with MDE and treated with tianeptine (from whom they were naïve for the present episode). Tianeptine was initiated for 79.5% of subjects (*N* = 2419 outpatients) and was prescribed as a switch from another antidepressant treatment for 18.8% of subjects (*N* = 573 outpatients) while this information was missing for 1.7% (*N* = 50). Dosage of tianeptine ranged between 12.5 and 37.5 mg/day, according to prescription recommendations. All other concomitant treatments for current somatic problems or associated symptoms of depression (e.g., sleep and agitation) were permitted, based on clinical judgment (see section “Treatments”). At the first visit, practitioners validated the diagnosis of MDE according to DSM-IV criteria. The practitioners also collected demographic data, major depressive disorder history, lifetime suicide attempt and alcohol or substance dependence at the first visit during a face-to-face interview. Non-inclusion criteria were age under 18 years old, non-Caucasian ethnicity, alcohol and substance dependence, or any other psychiatric pathology from axis I other than current MDE. The study was performed according to French regulatory guidelines and current codes of Good Clinical Practice. This study was approved by a local independent ethical committee (Comité de Protection des Personnes Ile de France XI/CPPIDF11, #08042). Each patient was informed about the aims and procedures of the study and provided written, signed consent.

Depression was assessed with a French version of the hospital anxiety and depression scale (HADS) 16 items at baseline V1. The second visit (V2) was followed in a window between 6 and 8 weeks and a novel evaluation of the HADS was done. Furthermore, outpatients made a self-evaluation of the HADS at V1 and V2 and at 15 and 30 days after the beginning of the treatment. This scale has a good performance assessing depression severity in both psychiatric and primary care patients, a good change-sensitivity^28^ and is simply to understand and use. Most factor analyses found a two-factor solution in accordance with the Anxiety (HADS-A) and Depression (HADS-D) subscales.

### Phenotype definition of treatment response

A positive treatment response (Resp+) was defined as the reduction of at least 50% of the HADS score at V2 compared to V1. Remission of depression was defined as a HADS score lower than a cutoff of 8 at V2. Finally, an early partial response was defined with a difference of reduction of the HADS higher than 25% after 2 weeks according to the report of the outpatient.

### Treatments

Associated treatments that may have an impact on treatment response (23 with antiparkinsonian drugs, 24 with corticosteroids, and 152 with thyroid hormones) were also recorded, but they had the same frequency in both response groups (*p* > 0.186).

On the other hand, 2.1% (*N* = 62) of outpatients also received a mood stabilizer, 2.7% (*N* = 83) a mood stabilizer and benzodiazepine, 50.1% (*N* = 1525) only benzodiazepine, and 45.1% (*N* = 1372) no mood stabilizer nor benzodiazepine. Patients with mood stabilizers, benzodiazepines, or both, had lower chances of treatment response, respectively, 39.3%, 41.6%, and 41.2% were responders versus 44.6% without (Chi² = 7.152, d.f. = 1, *p* = 0.007).

The average dosage of tianeptine was not equivalent in responders and non-responders, more patients with lower dosages being in the group of non-responders. Because the range of dosage was high (between 12.5 and 75 mg per day) and because the majority of patients were treated in accordance with guidelines (i.e. 37.5 mg a day for more than 82% in both groups), later analyses systematically checked the initial results restricting the analyses to patients treated with the recommended dosage of 37.5 mg/day.

### SNPs and genotyping

Genomic DNA from outpatients was extracted according to the protocol of the manufacturer from salivary sample collected with Oragene DNA kit (DNA Genotek Inc). A total of 16 SNPs encompassing four pharmacogenetics candidate genes (*NR3C1*, *FKPB5*, *CRHR1*, and *AVPR1B*) were screened using TaqMan SNP genotyping assays on an Applied Biosystem 7900HT Fast Real-Time PCR System (Life Technologies).

Sixteen SNPs were selected in the Hapmap database of tagged SNPs that cover the haplotype blocks across the genes, according to associations with antidepressant response or MDD.

### Statistical analysis

Hardy–Weinberg equilibrium tests, linkage disequilibrium *D*′ and *r*^2^ values and minor allele frequencies for the different SNPs were computed using Haploview 4.2 software.

Categorical variables were presented as percentages, and quantitative variables as means with standard deviation. Comparisons of sociodemographic, clinical features, and genotype between patients’ groups were performed using Student’s *t*-test (normality was tested with Shapiro–Wilk test) for continuous variables and chi-square test for categorical variables. Furthermore, logistic regression was carried out for sociodemographic and clinical features as intermediate variables with CRHR1 SNP rs878886 in predictor for treatment response. General linear models were computed to generate risk factors associated with the positive treatment response in interaction with or without the CRHR1 SNP rs878886 response allele. Those analyses were performed with SPSS statistical software (version 23; IBM SPSS Statistics for Windows. Armonk, NY, IBM Corp.).

We used a structural equation model with simple mediation, which assumes that the effect of sociodemographic characteristics and depressive disorder features on treatment response is the same in people with and without rs8788861. This was done in the goal to examine whether differences in the prevalence of sociodemographic characteristics and depressive disorder features, associated with rs8788861, explain the greater response to tianeptine among individuals with this SNP (see Supplementary Materials & Methods). All analyses were conducted in Mplus Version 7.2 (Muthen & Muthen). Mplus provides estimates and tests of significance for direct and specific effects and total indirect effects. The default estimator for the analysis was the variance-adjusted weighted least-squares, a robust estimator appropriate for categorical variables.

## Results

### Treatment response to antidepressant in outpatients with an MDE

A total of 3212 Caucasian outpatients were treated by tianeptine for an MDE (Table 1), including a majority of women (62.5%). At baseline (V1), the mean of HADS score was 27.8 and was decreased to 15.42 at the second visit (V2) after 43 ± 13 days.

**Table 1:** Sociodemographic and clinical characterictics according to tianeptine response in a sample of 3212 outpatients.

Subjects	Total (*N* = 3212)				Responder (*N* = 1298)				Non-responder (*N* = 1734)				Statistics			
	*N*	%	Average	s.d.	*N*	%	Average	s.d.	*N*	%	Average	s.d.	Chi²	*F*	d.f.	*p*
Male	1136	37.5			504	38.8			632	36.4						
Female	1896	62.5			794	61.2			1102	63.6			1.80		1	0.185
Age			49.40	14.81			48.04	14.50			50.50	15.02		20.16	1, 2980	**<0.001**
Adoption	24	0.8			8	0.6			16	0.9			0.840		1	0.359
Familial status	3030				1296				1734				6.94		3	0.074
School level over 12 years	1343	44.6			496	61.4			847	51.0			34.96		2	**<0.001**
Worker	1643	54.5			773	60.0			870	50.5			30.47		3	**<0.001**
First MDE	1660	54.7			792	61			868	50.1			35.99		1	**<0.001**
Number of previous MDE	1324	41.2	2.85	2.82	492	37.9	2.69	3.12	832	48.0	2.94	2.62		2.40	1, 1322	0.122
Age first MDE			36.20	14.21			35.49	13.35			36.62	14.68		1.91	1, 1295	0.167
Duration of previous MDE			32.93	27.01			30.43	22.5			34.47	29.34		6.42	1, 1210	**0.011**
Previous SA	363	12.3			118	9.3			245	14.5			18.20		1	**<0.001**
Number of previous SA			1.66	1.58			1.75	1.75			1.62	1.49		0.47	1, 361	0.49
Interval V1–V2 (days)			43.01	13.24			44.73	11.75			41.72	14.12		39.00	1, 3025	**<0.001**
V1-HAD-Anxiety			13.90	3.50			14.56	3.46			13.41	3.44		83.13	1, 3034	**<0.001**
V1-HAD-Depression			13.89	3.87			15.02	3.54			13.05	3.88		208.27	1, 3034	**<0.001**
V1-HAD-Total			27.80	6.11			29.59	5.74			26.46	6.04		208.51	1, 3034	**<0.001**

Significant *p* values are in bold.

Tianeptine was a treatment initiated in 79.5% of the MDE outpatients (*N* = 2419). A total of 42.8% outpatients were responders to tianeptine treatment. For the group of responders, the baseline of HADS score was 29.6 and decreased to 9.3 at V2, while in the group of non-responders, the V1 HADS score was 26.5 and decreased only to 20. MDE outpatients with mood stabilizers, benzodiazepines, or both were responders (respectively, 39.3%, 41.6%, and 41.2%) but they have significantly lower chances of treatment response versus 44.6% responders without mood stabilizer or benzodiazepines (*p* = 0.007).

Sex ratio was not different between responders and non-responders (Table 1). Responders were significantly younger than non-responders (48.04 ± 14.50 versus 50.50 ± 15.02 years), and they had a higher educational level and were more often currently working (Table 1). There were significantly more outpatients with a first MDE in the group of responders than in the non-responders group (61.0% versus 50.1%, *p* < 0.001). The duration of previous MDE was significantly lower in the group of responders (30.43 ± 22.50 months) compared to the group of non-responders (34.47 ± 29.34). Finally, the percentage of subjects who attempted suicide was significantly lower in the group of responders (Table 1).

### Pharmacogenetics association of CRHR1 with antidepressant response

A total of 16 SNPs encompassing the four candidate genes, *NR3C1*, *FKPB5*, *CRHR1*, and *AVPR1B*, were genotyped. The allelic distribution of SNPs was compared between responders and non-responders to identify a pharmacogenetics association to tianeptine antidepressant treatment (Table 2). Two SNPs, rs878886 and rs16940665, located in the same gene *CRHR1*, showed a statistical significant association with the antidepressant response (*p* < 0.001). Responders were more frequently patients carrying the C allele of rs878886 (OR = 1.26; 95% CI = [1.11; 1.44]). For the T allele of rs16940665, the OR associated with antidepressant response was of 1.34 (95% CI = [1.13; 1.60]). These associations remained significant after correction for multiple tests (rs878886 *p*_corrected_ = 0.006; rs16940665 *p*_corrected_ = 0.013) and a random split of the sample size (Supplementary Table 1).

**Table 2:** Pharmacogenetic association between SNPs of genes regulating the CRF system and response to antidepressant treatment.

			Responders		Non-responders		Statistics		
Gene	SNPs	Allele	*N*	%	*N*	%	Chi²	d.f.	*p*
NR3C1	rs33388	A	1123	51.2	1510	52.1	0.41	1	0.520
	rs4912905	G	1640	75.5	2265	77.6	2.96	1	0.085
	rs2963155	A	1756	78.5	2266	76.6	2.71	1	0.100
	rs41423247	G	1433	65.7	1900	65.4	0.68	1	0.794
	rs6189	G	2150	97.8	2907	98.0	0.24	1	0.627
	rs4607376	G	1170	53.4	1493	50.7	3.57	1	0.059
	rs12656106	G	1188	54.9	1579	54.5	0.07	1	0.791
FKBP5	rs3800373	T	1488	70.1	2022	71.3	0.98	1	0.322
	rs7757037	G	1187	54.0	1549	52.5	1.01	1	0.316
	rs737054	C	1610	72.3	2111	71.9	0.14	1	0.706
	rs1360780	C	1505	68.6	2040	69.6	0.57	1	0.452
	rs9470080	C	1464	67.1	1970	67.8	0.28	1	0.600
	rs6902321	T	1567	68.8	2093	68.4	0.12	1	0.726
CRHR1	rs878886	C	1710	78.2	2153	73.9	12.39	1	**<0.001**
	rs16940665	T	1106	79.5	1180	74.2	11.39	1	**<0.001**
AVPR1B	rs28632197	G	1885	88.7	2572	89.5	0.70	1	0.402

Significant *p* values are in bold.

The pharmacogenetics association confers an attributable risk of response of 16.35% and 20.32%, respectively, for rs878886*C and rs16940665*T alleles.

Pharmacogenetics analyze were also performed according to the self-report after 2 and 4 weeks of treatment. Only SNP rs878886 of *CRHR1* gene showed a significant association with a positive treatment response (*p* = 0.019 at 2 weeks; *p* = 0.039 at 4 weeks).

### Pharmacogenetic association of CRHR1 with improvement and remission

We analyzed the pharmacogenetics to remission for tianeptine antidepressant treatment. Again, only the two SNPs located in the *CRHR1* gene showed a significant association (rs878886 Chi² = 8.482, *p* = 0.004; rs16940665 Chi² = 6.202, *p* = 0.013) (Supplementary Table 2). The OR with the C allele of rs878886 was of 1.32 (95% CI = [1.10; 1.60]) and for the T allele of rs16940665, the OR was of 1.36 (95% CI = [1.17; 1.73]). The pharmacogenetics association confers an attributable risk of remission of 19.52% and 21.29%, respectively, for rs878886*C and rs16940665*T alleles.

### CRHR1 haplotype association with response and remission to antidepressant

The variants rs878886 and rs16940665 were located in the same haplotype block (linkage disequilibrium of 0.99 and *r*^2^ of 0.99). The frequent haplotype rs878886*C–rs16940665*T was detected in 75.7% of the subjects. This haplotype was over-represented in 78.1% of responders compared to 73.9% of non-responders (Chi² = 12.327, *p* = 4.10–4). The OR for haplotype rs878886*C–rs16940665*T was of 1.26 (95% CI = [1.11; 1.43]). This haplotype confers an attributable risk of pharmacogenetics response of 16.23%.

The haplotype rs878886*C–rs16940665*T was also significantly over-represented in the remitters (79.8%) compared to the non-remitters (75.0%) (Chi² = 8.559, *p* = 0.003), conferring an OR = 1.32 (95% CI = [1.10; 1.60]) and an attributable risk of 19.48%.

### Is the involved SNP associated with treatment response through specific clinical features?

To identify an association between SNPs and treatment response through a specific sociodemographic or clinical features we performed logistic regression and ANCOVA. Thus, sociodemographic young age, low school level and worker status, as well as clinical features of a first MDE, young age of the first MDE and high score to the HADS were significantly associated with treatment response (Table 3).

**Table 3:** Logistic regression of socio and clinical traits and their potential role as intermediate variables with *CRHR1* SNP rs878886 in predictor for treatment response.

Covariable	Association with treatment response			Association with CRHR1 SNP rs878886			Interaction with CRHR1 to predict TTT response		
	Wald	*p*	OR	Chi²	*p*	OR	Chi²	*p*	OR
Gender: woman	1.19	0.28	n.a.^a^	11.56	**0.001**	0.75 (0.63–0.88)	1.13	0.29	n.a.
Age	21.73	**<0.001**	1.02(1.01–1.03)	3.33	0.068	0.65 (0.41–1.00)	8.13	**0.004**	0.99 (0.98–0.99)
Adoption	0.97	0.33	n.a.	12.32	**<0.001**	0.80 (0.69–90)	1.15	0.28	n.a.
Familial status: not alone	2.06	0.15	n.a.	1.06	0.30	n.a.	0.01	0.98	n.a.
School level over 12 years: low	9.43	**0.002**	1.55 (1.17–2.05)	7.56	**0.006**	0.71(0.56–0.91)	0.03	0.87	n.a.
Worker: yes	9.13	**0.003**	0.59 (0.42–0.83)	3.55	0.06	0.72 (0.51–1.01)	2.32	0.13	n.a.
First MDE	26.71	**<0.001**	0.54 (0.43–0.68)	13.46	**<0.001**	0.69 (0.56–0.84)	3.97	**0.046**	1.31 (1.00–1.71)
Number of previous MDE	0.71	0.40	n.a.	6.30	**0.012**	0.69 (0.51–0.91)	0.04	0.85	n.a.
Age at first MDE	4.74	**0.029**	1.01 (1.00–1.03)	0.01	0.94	n.a.	1.34	0.25	n.a.
Lifetime being depressed	0.36	0.55	n.a.	6.77	**0.009**	0.64 (0.46–0.90)	0.60	0.44	n.a.
Previous SA	0.23	0.63	n.a.	15.95	**<0.001**	0.75 (0.65–0.87)	4.56	0.03	1.60 (1.04–2.45)
Number of previous SA	1.28	0.26	n.a.	0.19	0.67	n.a.	0.02	0.88	n.a.
Interval V1–V2 (days)	6.27	**0.01**	0.99 (0.98–0.99)	0.16	0.69	n.a.	2.28	0.13	n.a.
V1-HAD-Anxiety	41.55	**<0.001**	0.89 (0.86–0.93)	1.45	0.23	n.a.	0.20	0.65	n.a.
V1-HAD-Depression	81.68	**<0.001**	0.86 (0.83–0.89)	1.20	0.27	n.a.	0.10	0.75	n.a.
V1-HAD-Total	89.07	**<0.001**	0.90 (0.88–0.92)	1.99	0.16	n.a.	0.68	0.41	n.a.

^a^Not applicable.

Significant *p* values are in bold.

Otherwise, female gender, adoption status, low school level, first MDE, number of previous MDE, lifetime MDE, and previous suicide attempt(s) were associated with *CRHR1* SNP rs878886.

Finally, significant interactions in responders and allele C of *CRHR1* rs878886 were observed for young age and first MDE, respectively, *p* = 0.004 and *p* = 0.046 (Table 3). While we observed a direct and strong effect in the positive treatment response and remission associated with the presence of the allele C of rs878886 or allele T of rs16940665, we cannot exclude direct and specific effects of sociodemographic status and/or clinical features (Supplementary Fig. 1). Thus, we investigate the simple mediation effect by structural equation models.

### Mediation model of joint effect on response to tianeptine of differences in sociodemographic characteristics and depressive disorder features associated with rs8788861

**Fig. 1:**
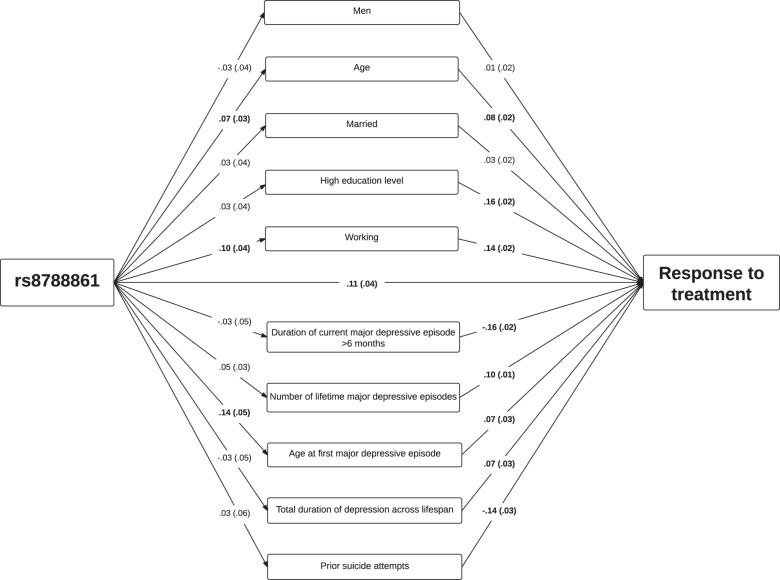
Mediation model of the relationship between rs8788861, differential associations with sociodemographic characteristics and depressive disorder features, and response to tianeptine. Regression coefficients are standardized. Values in brackets are standard errors. All coefficients in bold are significant (two-sided *p* < 0.05).

When examining all sociodemographic characteristics and depressive disorder features while mutually adjusting for each other in the same model, individuals with rs8788861 were more likely to be older at the time of the study and at the time of the first MDE. The response to antidepressant varied across sociodemographic characteristics and depressive disorder features: low education level, duration of current MDE of more than 6 months, and lifetime suicide attempts having the greater reducing effects on treatment response (Fig. 1).

The total indirect effect (obtained by summing the product of the effects of rs8788861 on each sociodemographic characteristic and depressive disorder feature with their effects on response to antidepressant) was not significant (*β* = 0.04, *p* = 0.07). This non-significant indirect effect implies that differences in prevalence of sociodemographic characteristics and depressive disorder features associated with rs8788861 do not explain the greater response to tianeptine among individuals with rs8788861.

The model also found a significant positive direct effect (i.e., an effect not due to the differences in sociodemographic characteristics and depressive disorder features) of rs8788861 on response to tianeptine (standardized *β* = 0.11, *p* = 0.011), suggesting that the effect of at least certain sociodemographic characteristics and depressive disorder features on response to tianeptine could differ between individuals with and without rs8788861. The sensitivity analysis excluding participants with missing data yielded similar results with a total indirect effect not significant (standardized *β* = 0.01, *p* = 0.675) and a significant positive direct effect of rs8788861 (standardized *β* = 0.14, *p* = 0.037) (Supplementary Fig. 2).

The results of this model, particularly the direct effect of rs8788861 on response to tianeptine, suggested that the mediational model did not explain totally the greater response to tianeptine among individuals with rs8788861.

### Moderated mediation model of joint effect on response to tianeptine of differences in frequency and effect of sociodemographic characteristics and depressive disorder features associated with rs8788861

**Fig. 2:**
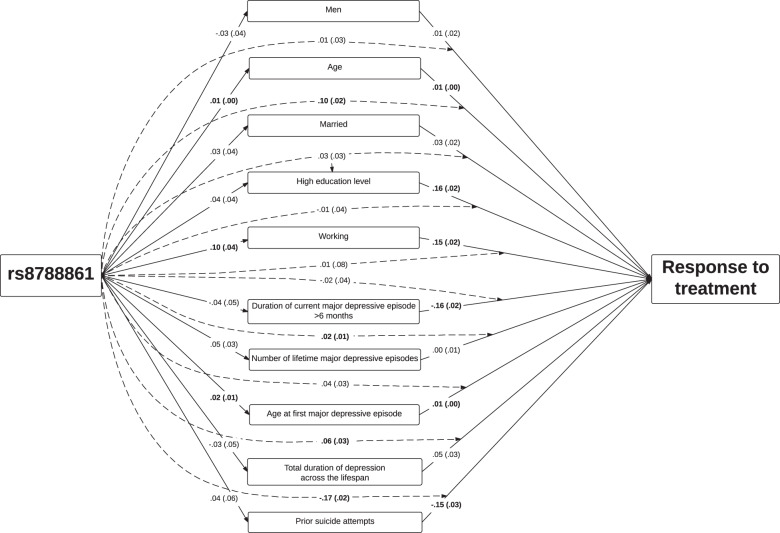
Moderated mediation model of the effects of rs8788861 and sociodemographic characteristics and depressive disorder features on response to tianeptine. Regression coefficients are standardized. Values in brackets are standard errors. All coefficients in bold are significant (two-sided *p* < 0.05). Dotted arrows indicate moderation effects of rs8788861 on the relationships between sociodemographic characteristics and depressive disorder features and response to tianeptine), e.g. the reducing effect of lifetime history of suicide attempt on response to tianeptine is greater in depressed individuals without rs8788861 than in those with rs8788861.

When allowing the effects of sociodemographic characteristics and depressive disorder features to be moderated by rs8788861, there were significant interactions between rs8788861 and age, duration of current MDE higher than 6 months, age at first MDE, and history of suicide attempts. The strongest moderating effect of rs8788861 was on history of suicide attempts and the weakest effects were on sex, working status, and education level (Fig. 2). The total indirect effects in this model was *β* = 0.23 (SE = 0.08, *p* < 0.01) and the residual direct effect of rs8788861 was not significant (*β* = −0.09, SE = 0.09, *p* = 0.324), indicating that the total effect of rs8788861 on response to tianeptine (i.e. *β* = 0.15, SE = 0.04) was well explained by this moderated mediation model. The sensitivity analysis excluding participants with missing data yielded similar results, with total indirect effects (*β* = 0.10, SE = 0.05, *p* < 0.01) and a non-significant residual direct effect of rs8788861 (*β* = −0.04, SE = 0.18, *p* = 0.835) (Supplementary Fig. 3).

Thus, this model indicated that differences in the effects of sociodemographic characteristics and depressive disorder features explained a substantial proportion of differences in response to tianeptine associated with rs8788861.

## Discussion

In this study, we have investigated the pharmacogenetics association of multiple variants encompassing four genes involved in the HPA axis, including *NR3C1*, *FKPB5*, *CRHR1,* and *AVPR1B*, in a large population of more than 3200 Caucasian outpatients harboring an MDE, recruited and followed up for at least 6 weeks and receiving the atypical antidepressant tianeptine, initiated in 79.5% of the outpatients and change in 18.8%. Outpatients with additional treatments, including antiparkinsonian drugs, corticosteroids, thyroid hormones, mood stabilizers, antipsychotics, or both, do not have an advantage in treatment response compared to responders without these treatments. Only two polymorphisms within the *CRHR1* gene, rs878886 and rs16940665, and haplotype rs878886*C–rs16940665*T were found significantly associated with a better response to tianeptine, as well as a better remission.

Variants of the *CRHR1* gene could be intermediate factors between sociodemographic features or clinical characteristics and treatment response. To avoid this, we recruit outpatients in real life and followed them up for at least 6 weeks, with HADS self-reports at 2- and 4 weeks in addition to HADS reports completed by practitioners at baseline and at the end of follow-up. When sociodemographic features are taken into account, we found common features associated with a better treatment response: a young age, a higher educational level, and a job situation (Table 1). The same observations were done for the clinical characteristics: a first MDE, a lower duration of previous MDE, and less lifetime suicide attempts in the group of responders compared to the non-responders. When we added the *CRHR1* variant rs878886, all these characteristics have less impact in the treatment response and remission than the fact to carry the C antidepressant responder allele.

The problem of chance finding is crucial in pharmacogenetic studies according of the numerous discrepancies found in different publications and the possible clinical heterogeneity of the patients, especially these with an MDE. Accordingly, we used three different technics in order to potential reduce this risk. Firstly, we applied the Bonferroni correction although sometimes considered as too conservative, i.e. dividing the *p* value by the number of SNPs (rs878886 *p*_corrected_ = 0.006; rs16940665 *p*_corrected_ = 0.013). Secondly, we focused not only on treatment response but also we assessed remission, two concepts that are overlapping but nevertheless not identical (rs878886 *p* = 0.004; rs16940665 *p* = 0.013). And lastly, thanks to a large sample size of 3212 MDE outpatients, we randomly split the cohort in two subsamples and confirmed that our results were independently detected (Supplementary Table 1).

Several independent studies have reported pharmacogenetics associations between different polymorphisms of *CRHR1* gene with response and remission for several antidepressants, including SSRIs (fluoxetine and citalopram) or a TCA (desipramine)^14–16^. These observations were found in small cohorts of few hundreds to up to two thousands (i.e. STAR*D) depressed patients, from different ethnic origins, and under different antidepressant treatments. In contrast, some other studies did not reported pharmacogenetics association between different *CRHR1* polymorphisms and antidepressant treatments^29,30^. Thus, no associations with *CRHR1* variants were observed in 159 depressive outpatients treated by citalopram (SSRI) during 4 weeks^30^. Furthermore, the sequencing of *CRHR1* in 272 unrelated Mexican Americans with MDE allowed to identify a total of 56 polymorphisms among the gene, including 23 new variants and one that changes the amino acid sequence of the protein. However, none of them were associated with an antidepressant treatment response^31^. Finally, in a 6-week study in duloxetine treated (a serotonin norepinephrine reuptake inhibitor) of 241 MDE patients, only rs4792888 of *CRHR1* showed a trend of pharmacogenetics association (*p* = 0.054)^32^. These discrepancies could be due to the involvement of different polymorphisms located in other genes in the biological pathway of the HPA axis or they could depend on mechanism of action of antidepressant used.

Thus, polymorphisms in the genes *FKBP5* and *NR3C1* were found to be associated with antidepressant response in independent studies^11–13,17^. In the present study, we did not observed a pharmacogenetics association for *FKBP5* and *NR3C1* genes, may be due to the type of antidepressant used or to the outpatients sample. Other variants of genes involved in the HPA axis, including *CRHR2* and *CRHBP*, have been reported associated with antidepressant, mostly SSRI or TCA, response or remission^15–17^. In a recent work, the analysis of 636 patients from the International Study to Predict Optimized Treatment in Depression (iSPOT-D), treated 8-week with antidepressants escitalopram (SSRI), sertraline (SSRI), or extended-release venlafaxine (SNRI), have showed a pharmacogenetics association with *CRHBP* variant rs28365143^33^. Again, discrepancies have been observed between pharmacogenetics association and these genes involved in the biological pathway of the HPA axis.

These divergent observations could be due to different clinical characteristics or different activity of the HPA axis in patients. Thus, it has been reported that fluoxetine (SSRI) responders and remitters exhibited significantly lower circadian cortisol levels than those who did not respond. Furthermore, MDD treated patients who abandoned before the third week presented a low cortisol levels^29^. Several studies have reported that variants in *CRHR1* have been associated with specific cortisol responses and stress-system processes^32–35^. While other works have supported that polymorphisms of other genes of CRH axis, like *CRHBP* gene, affect the cortisol responses^36,37^. Variants in *CRHR1* gene have been found associated with several clinical features, like stress, panic, emotion, work memory, and disorders including MDE but also general anxiety disorder^37–42^. Furthermore, these association have been found also in a strong interaction with early life stress and childhood maltreatment^41–43^. In our study, early adverse events have not been collected. Because early adverse events were not collected, we could not assess their differences between responders and non-responders, which represents a limit of the present work. Interestingly, alleles of rs878886 of *CRHR1* gene have been associated with fear acquisition in humans^44,45^. These studies showed that G-allele carriers have a reduced acquisition of cue-specific fear-conditioned responses compared to C/C homozygotes. The cued-fear acquisition deficits is also under the genetic interaction of variations of the *SL6A4/5HTT* gene, which codes for the serotonin transporter^44,45^. This fear acquisition under the control of *CRHR1* and *SLC6A4* variants may be also a limitation of this study due to the impact of fear control between the responders and non-responders. These implications, direct or indirect, of *CRHR1* variants in the clinical features and disorders, in interaction with life events, can modulate the clinical phenotypes of the MDE subjects. And these *CRHR1* variants can also impact, directly or indirectly, antidepressant treatment response.

Here, we investigate the pharmacogenetics treatment response of the atypical antidepressant tianeptine. Tianeptine enhances a cascade of cellular adaptations that prevents and reverses the dendritic remodeling induced by the stress or glucocorticoids and increases dendritic length and branching of neurons in the amygdala^19,20^. Interestingly, it has been showed that serotonin from the dorsal raphe nucleus enhances fear and anxiety and activates a subpopulation of corticotropin-releasing factor neurons in the bed nucleus of the stria terminalis^46^. This circuit could be modulated by tianeptine to improve a better adaptation, in contrast to the clinical observations of early adverse events to SSRI treatment in some patients. Another propriety of tianeptine is its MOR agonist that acts specifically on the opioidergic system^22^. Indeed, a recent study has shown, in mice model, that the antidepressant- and opioid-like behavioral effects of tianeptine were mediated by its MOR agonist^22^. Interestingly, cortisol response is modulated by the endogenous opioid, beta-endorphin, via the MOR and variant in the MOR gene *OPRM1* affects the cortisol stress response^23^ and, as mentioned previously, opioid system partly regulates HPA axis activation.

To conclude, our study was able to strongly replicate a pharmacogenetics association of an atypical antidepressant tianeptine treatment for MDE, with one of the gene involved in the HPA axis, *CRHR1* gene. This pharmacogenetics work shows *CRHR1* association with (1) more specifically attributed to treatment response, (2) with a lower risk of chance finding, and (3) in a more ecological situation. Our results should encourage studies on the link between opioid system and HPA axis. Furthermore, recent findings on ketamine support an interaction between these systems. Indeed, it has been shown that ketamine increases the effectiveness of the signaling induced by opiates^47^ and in a very recent randomized controlled trial^48^, they found that reduction of depression was lower when naltrexone (an MOR antagonist), versus placebo, was administered before ketamine administration. Regarding those data and results of our study, it seems that there is a real interaction between HPA and opioid systems and this interaction may modulate response to antidepressant treatment and evolution of depression.

## Supplementary information

Supplementary Materials And Methods

Pharmacogenetic association in 2 random samples of the splited GENESE population between SNPs of genes regulating the CRF system and response or remission to antidepressant treatment

Pharmacogenetic association between SNPs of genes regulating the CRF system and remission to antidepressant treatment

Impact of pharmacogenetics variant rs878886*C on sociodemographic and clinical features and interactions to the treatment response

Mediation model of the relationship between rs8788861, differential associations with sociodemographic characteristics and depressive disorder features, and response to tianeptine in individuals without missing data

Moderated mediation model of the effects of rs8788861 and sociodemographic characteristics and depressive disorder features on response to tianeptine in individuals without missing data
