# Supplementary material for: Corticotropin releasing hormone receptor CRHR1 gene is associated with tianeptine antidepressant response in a large sample of outpatients from real-life settings
Source: Transl Psychiatry. 2020 Nov 5;10:378. doi: 10.1038/s41398-020-01067-y (PMC7644692; doi:10.1038/s41398-020-01067-y)
Supplement: Supplementary file 1 — Supplementary Materials And Methods [file 41398_2020_1067_MOESM1_ESM.docx]

**Supplementary Materials And Methods**

The mediation model allows for the possibility of compensatory effects (i.e. that some indirect effects can be positive while others can be negative). The mediation model would explain the greater response rate of individuals with rs8788861 if the total indirect effect (i.e. the sum of all the specific indirect effects of rs8788861 on treatment response through all sociodemographic characteristics and depressive disorder features) would be positive and there would be no additional (i.e. direct) effect of rs8788861 on response rate. By contrast, a null total indirect effect and the presence of a direct effect would suggest the existence of differential effects of sociodemographic characteristics and depressive disorder features on the likelihood of response (i.e. a moderating effect of rs8788861 on likelihood of response from different sociodemographic characteristics and depressive disorder features). To test the presence of this moderating effect while simultaneously taking into account differences in the prevalence of sociodemographic characteristics and depressive disorder features associated with rs8788861, we used a structural equation model with moderated mediation.^1-4^  This model incorporated a moderating effect of rs8788861 on the relationship of each sociodemographic characteristic and depressive disorder feature with response to tianeptine, modeled as an interaction between rs8788861 and the response to tianeptine conditional on the sociodemographic characteristic or the depressive disorder feature.

Because we sought to examine simultaneously all path coefficients, no paths in any of the models were fixed to zero. Therefore, goodness of fit measures are not relevant in evaluating these models since they do not inform on the ‘correctness’ of the models but rather provide only a summary of how well the observed correlations match the model when several paths are fixed at zero.^5^ Missing data were imputed using Markov chain Monte Carlo (MCMC) methods^6^. To examine the robustness of our results, we performed sensitivity analyses while excluding participants with any missing data.

All analyses were conducted in Mplus Version 7.2^7^. Mplus provides estimates and tests of significance for direct effects and specific and total indirect effects. The default estimator for the analysis was the variance-adjusted weighted least squares (WLSMV), a robust estimator appropriate for categorical variables.

**Supplementary References**

1. Preacher KJ, Rucker DD, Hayes AF. Addressing moderated mediation hypotheses: Theory, methods, and prescriptions. Multiv Behav Res. 2007;42:185-227.

2. MacKinnon DP. Contrasts in multiple mediator models. In J. S. Rose, L. Chassin, C. C. Presson, & S. J. Sherman (Eds.), Multivariate applications in substance use research (pp. 141–160). Mahwah, NJ: Lawrence Erlbaum Associates, Inc; 2000.

3. MacKinnon DP. Commentary on Donaldson, Mediator and moderator analysis in program development. In S. Sussman (Ed.), Handbook of program development for health behavior research and practice (pp. 497–500). Thousand Oaks, CA: Sage; 2001.

4. Preacher KJ, Curran PJ, Bauer DJ. Computational tools for probing interaction effects in multiple linear regression, multilevel modeling, and latent curve analysis. J Educational Behav Statistics. 2006;31:437-48.

5. Stage FK, Carter H, Nora A. Path Analysis: An Introduction and Analysis of a Decade of Research. J Educational Res. 2004;98:5-12.

6. Schafer JL. Analysis of Incomplete Multivariate Data. New York: Chapman and Hall; 1997.

7. Muthen LK, Muthen BO. Mplus User’s Guide Los Angeles; 1998-2006.
