# Supplementary material for: Corticotropin releasing hormone receptor CRHR1 gene is associated with tianeptine antidepressant response in a large sample of outpatients from real-life settings
Source: Transl Psychiatry. 2020 Nov 5;10:378. doi: 10.1038/s41398-020-01067-y (PMC7644692; doi:10.1038/s41398-020-01067-y)
Supplement: Supplementary file 2 — Pharmacogenetic association in 2 random samples of the splited GENESE population between SNPs of genes regulating the CRF system and response or remission to antidepressant treatment [file 41398_2020_1067_MOESM2_ESM.pdf]

Supplementary Table 1: Pharmacogenetic association in 2 random samples of the splited GENESE population between SNPs of genes regulating the CRF system and response or remission to antidepressant treatment

			RANDOM Sample1 N=1606		RANDOM Sample2 N=1606	
Gene	SNPs	Allele	P value Resp+	P value Rem+	P value Resp+	P value Rem+
NR3C1	rs33388	A	0.927	0.839	0.418	0.983
	rs4912905	G	0.252	0.544	0.192	0.432
	rs2963155	A	0.508	0.288	0.094	0.418
	rs41423247	G	0.776	0.793	0.501	0.840
	rs6189	G	0.975	0.224	0.509	0.431
	rs4607376	G	0.430	0.549	0.058	0.025
	rs12656106	G	0.817	0.481	0.900	0.386
FKBP5	rs3800373	T	0.444	0.646	0.523	0.856
	rs7757037	G	0.604	0.497	0.051	0.096
	rs737054	C	0.260	0.698	0.094	0.736
	rs1360780	C	0.619	0.793	0.555	0.946
	rs9470080	C	0.707	0.988	0.701	0.953
	rs6902321	T	0.930	0.983	0.572	0.991
CRHR1	rs878886	C	**0.010**	0.173	**0.015**	**0.006**
	rs16940665	T	**0.011**	0.241	**0.024**	**0.019**
AVPR1B	rs28632197	G	0.233	0.127	0.985	0.618
