# Supplementary material for: Corticotropin releasing hormone receptor CRHR1 gene is associated with tianeptine antidepressant response in a large sample of outpatients from real-life settings
Source: Transl Psychiatry. 2020 Nov 5;10:378. doi: 10.1038/s41398-020-01067-y (PMC7644692; doi:10.1038/s41398-020-01067-y)
Supplement: Supplementary file 3 — Pharmacogenetic association between SNPs of genes regulating the CRF system and remission to antidepressant treatment [file 41398_2020_1067_MOESM3_ESM.pdf]

Supplementary Table 2: Pharmacogenetic association between SNPs of genes regulating the CRF system and remission to antidepressant treatment

			Remitters		Non-remitters		Statistics		
Gene	SNPs	Allele	N	%	N	%	Chi²	df	p
NR3C1	rs33388	A	402	51.5	2245	51.8	0.02	1	0.907
	rs4912905	G	577	75.3	3348	76.9	0.937	1	0.331
	rs2963155	A	612	77.7	3433	77.5	0.016	1	0.926
	rs41423247	G	505	65.4	2843	65.5	0.002	1	0.967
	rs6189	G	767	98.6	4316	97.8	1.902	1	0.217
	rs4607376	G	432	55.1	2241	51.3	3.839	1	0.051
	rs12656106	G	407	52.9	2373	55.0	1.190	1	0.289
FKBP5	rs3800373	T	528	70.2	3005	71.0	0.195	1	0.663
	rs7757037	G	432	55.8	2315	52.6	2.707	1	0.101
	rs737054	C	564	71.9	3173	72.0	0.002	1	0.966
	rs1360780	C	535	68.9	3033	69.3	0.042	1	0.833
	rs9470080	C	523	67.9	2933	67/9	0.000	1	1.000
	rs6902321	T	552	68.7	3130	68.7	0.000	1	1.000
CRHR1	rs878886	C	615	79.9	3268	75.0	8.482	1	**0.004**
	rs16940665	T	408	81.0	1880	75.8	6.202	1	**0.013**
AVPR1B	rs28632197	G	667	88.5	3812	89.3	0.48	1	0.484
