# Supplementary figures and images for: Corticotropin releasing hormone receptor CRHR1 gene is associated with tianeptine antidepressant response in a large sample of outpatients from real-life settings
Source: Transl Psychiatry. 2020 Nov 5;10:378. doi: 10.1038/s41398-020-01067-y (PMC7644692; doi:10.1038/s41398-020-01067-y)

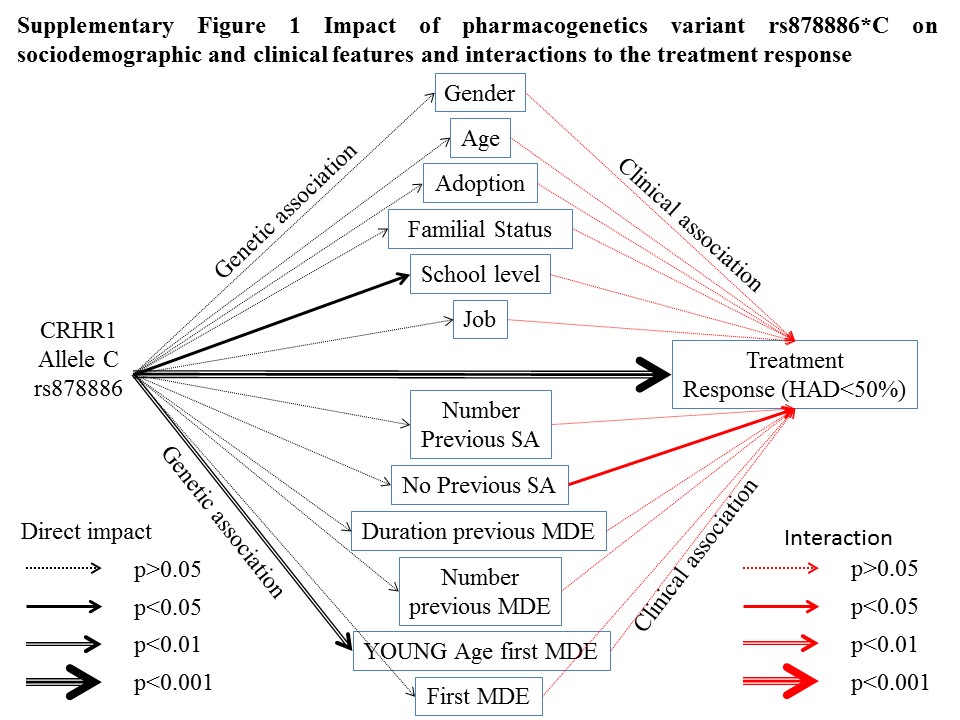

Supplement: Supplementary file 4 — Impact of pharmacogenetics variant rs878886*C on sociodemographic and clinical features and interactions to the treatment response [file 41398_2020_1067_MOESM4_ESM.jpg]

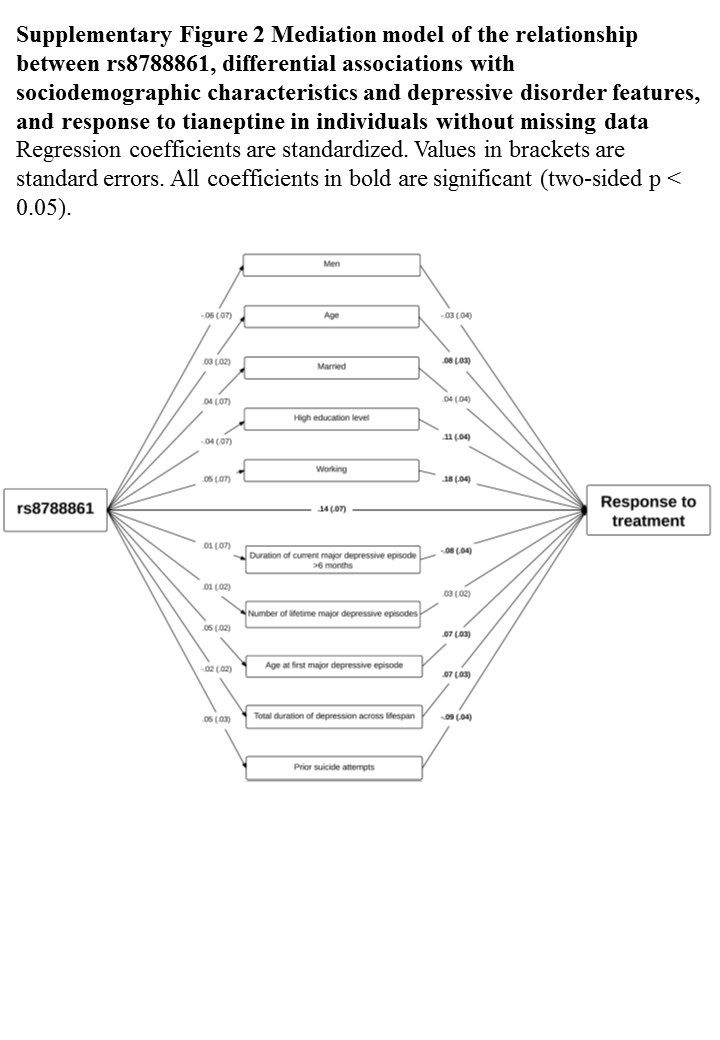

Supplement: Supplementary file 5 — Mediation model of the relationship between rs8788861, differential associations with sociodemographic characteristics and depressive disorder features, and response to tianeptine in individuals without missing data [file 41398_2020_1067_MOESM5_ESM.jpg]

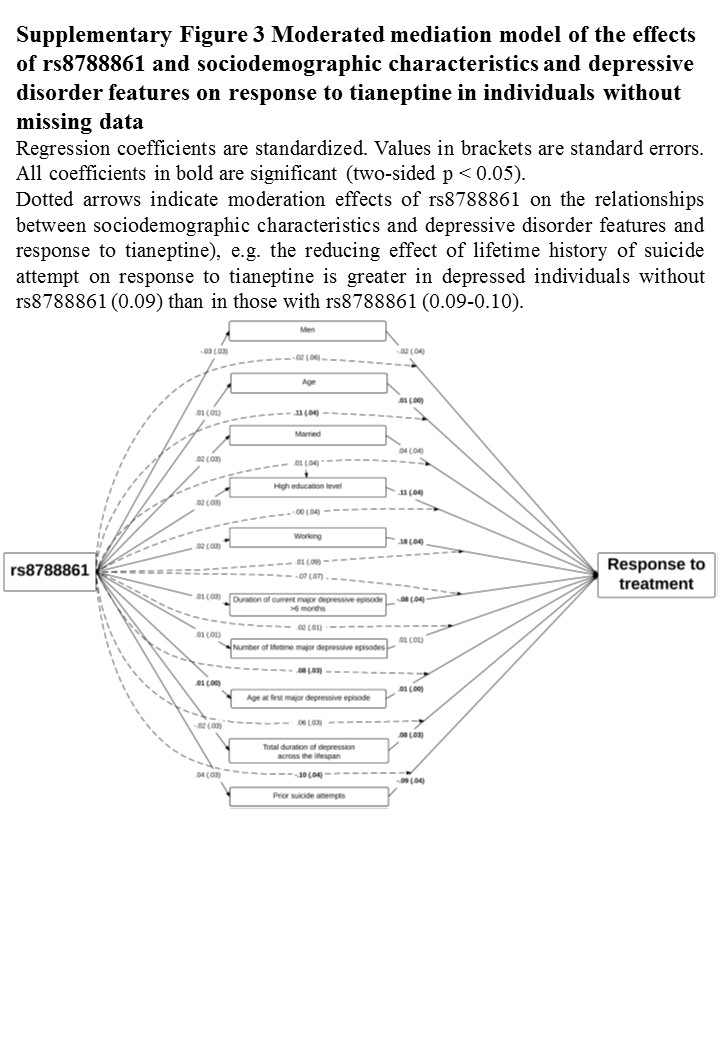

Supplement: Supplementary file 6 — Moderated mediation model of the effects of rs8788861 and sociodemographic characteristics and depressive disorder features on response to tianeptine in individuals without missing data [file 41398_2020_1067_MOESM6_ESM.jpg]
